# Laboratory Exposures from an Unsuspected Case of Human Infection with *Brucella canis*

**DOI:** 10.3201/eid2709.204701

**Published:** 2021-09

**Authors:** Jasmine Ahmed-Bentley, Susan Roman, Yazdan Mirzanejad, Erin Fraser, Linda Hoang, Edward J. Young, Muhammad Morshed, Gregory Deans

**Affiliations:** Fraser Health, Surrey, British Columbia, Canada (J. Ahmed-Bentley, S. Roman, Y. Mirzanejad, G. Deans);; University of British Columbia, Vancouver, British Columbia, Canada (J. Ahmed-Bentley, Y. Mirzanejad, E. Fraser, L. Hoang, M. Morshed, G. Deans);; British Columbia Center for Disease Control Public Health Laboratory, Vancouver (E. Fraser, L. Hoang, M. Morshed);; Baylor College of Medicine, Houston, Texas, USA (E.J. Young)

## Abstract

We report a case of human infection with a *Brucella canis* isolate in an adult in Canada who was receiving a biologic immunomodulating medication. We detail subsequent investigations, which showed that 17 clinical microbiology staff had high-risk exposures to the isolate, 1 of whom had a positive result for *B. canis*.

A 70 year-old woman came to the emergency department at Abbotsford Regional Hospital (Abbotsford, BC, Canada) after 3 days of chills, headache, nausea, weakness, and urinary frequency. Her medical history included psoriasis and psoriatic arthritis; her medications included ixekizumab. Her vital signs were within reference limits. Initial blood test results were within reference ranges, apart from a mild increase in the monocyte level (0.9 × 10^9^ cells/L, reference range 0.1‒0.8 × 10^9^ cells/L) and a high level of C-reactive protein (14.5 mg/L, reference value <7.5 mg/L). Chest radiograph showed no acute findings. The patient, believed to have a urinary tract infection, was discharged and given a 7-day course of oral cefixime.

Four BacT/Alert blood culture bottles (bioMérieux, https://www.biomerieux-usa.com) (3 aerobic and 1 anaerobic) were collected in the emergency department. One aerobic blood culture bottle showed a positive result for *Brucella* sp. after 3.5 days of incubation. A laboratory technologist prepared a slide for Gram staining and subcultured a sample into medium in a biosafety cabinet while wearing a gown and gloves. The result of the Gram stain was difficult to interpret and was initially reported as showing gram-positive cocci. A urine culture result was negative. The patient was readmitted to the emergency department for reassessment. She reported feeling somewhat better and continued using the oral antimicrobial drug while awaiting further information from the laboratory.

Culture plates were examined for growth every 4 hours and after 42 hours of incubation showed faint growth on blood and chocolate agars. Matrix-assisted laser desorption/ionization time-of-flight (MALDI-TOF) mass spectrometry (Bruker Daltonics Instrument; https://www.bruker.com) did not identify the organism, but the highest score was for *Ochrobactrum* spp. At this point, culture examinations and MALDI-TOF mass spectrometry target plate preparations were performed on an open laboratory bench.

The next day, after reviewing the Gram stain result and MALDI-TOF mass spectrometry results, a microbiologist noted that the organisms were pleomorphic, gram-negative coccobacilli, which increased the possibility of *Brucella* spp. The provincial reference laboratory (British Columbia Centre for Disease Control Public Health Laboratory, Vancouver, BC, Canada) was contacted. That evening, molecular testing at that laboratory confirmed the isolate was *Brucella* spp. and the isolate was sent to the National Microbiology Laboratory (Winnipeg, MB, Canada) for species identification. The microbiologist communicated this result to the emergency physician, public health officials, and laboratory leadership; the patient was contacted and returned the same day to initiate outpatient therapy (intravenous gentamicin and oral doxycycline). After 7 days of therapy, the patient was much improved; gentamicin was discontinued, and the patient was transitioned to receiving oral rifampin and doxycycline for 6 weeks of treatment. Blood cultures repeated on treatment day 7 had no growth. No focal site of infection was identified after analysis of medical history, physical examination, and computed tomography of the chest, abdomen, and pelvis.

The patient reported a major headache and a depressed mood as she neared the tentative end of her treatment; therapy was extended while investigating for evidence of neurobrucellosis, which would require further prolongation of therapy. Computed tomography of the head and lumbar puncture found no evidence of central nervous system infection; thus, treatment was discontinued (53 days of completed total therapy). The headache and mood changes for the patient resolved within days of treatment discontinuation, and she did not have any symptoms of recurrence after 1 year.

The National Microbiology Laboratory reported the identification as *B. canis*. A public health investigation determined that the patient had helped transport rescue dogs from Mexico and the United States to Canada (*1*). Ten weeks before this patient’s onset of symptoms, a pregnant dog from Mexico spontaneously aborted 2 stillborn puppies in the patient’s car. After *B. canis* was identified in the human patient, testing of the dog showed that it was positive for *B. canis* by immunofluorescent antibody test. We conducted outreach for *B. canis* detection, prevention, and control of the dog rescue organization and to veterinary and medical professionals in British Columbia.

The patient was seronegative for *B. abortus* (by microagglutination test) and seropositive for *B. canis* (by D-TEC CB Commercial Slide Agglutination Kit; Zoetis, https://www.zoetisus.com). Serologic testing for *B. canis* was performed at Baylor College of Medicine (Houston, TX, USA). This kit is intended for veterinary use; the sensitivity and specificity for human samples is unknown because there are few cases of human *B. canis* infection in North America. Although this kit is not validated for human samples, anecdotal evidence suggests this test provides results that correlate with the clinical picture (E.J. Young, unpub. data). Also, serologic agglutination assays using *B. abortus* antigens do not cross-react with antibodies to *B. canis* (*2*).

As part of the laboratory exposure investigation, we reviewed the workup in the microbiology laboratory and the location of all personnel to identify potential high-risk exposures (<5 feet from culture manipulation on an open bench) (*3*). A microbiologist performed a risk assessment for all exposed staff (*3*). No aerosol-generating procedures had been performed.

A total of 17 staff had high-risk exposures: 9 were technologists who worked directly on the *Brucella* culture on an open bench and 8 were staff who worked within a 5-foot radius. These staff were referred to an infectious diseases clinic for urgent assessment and consideration of postexposure prophylaxis. Serologic testing for *B. canis* and *B. abortus* was performed at 3 months and 6 months after exposure. One staff member had a positive result for *B. canis* that was detected at 3 months despite taking 3 weeks of postexposure prophylaxis initiated 12 days after a high-risk exposure. All other staff were seronegative. No exposed staff reported symptoms of *Brucella* infection during the 6 months of postexposure follow-up.

This case prompted the following procedure changes to prevent future laboratory exposures. First, *Brucella* spp. are aerobes typically requiring >48 hours to grow in automated blood culture systems because the level of bacteremia is usually low (1–5 CFU/mL), doubling time is long (2.5–3.5 hours), and CO_2_ production is low (*4*–*7*). Aerobic blood culture bottles showing a positive result after >48 hours of incubation are now processed by using additional personal protective equipment (N95 mask, face shield, gown, and gloves) in a biosafety cabinet, and subculture plates are labeled as containing a possible Risk Group 3 agent. A microbiologist or senior technologist reviews the Gram stain results and guides further workup.

Second, a security-relevant bacterial database was installed on the Bruker instrument. This database contains 1 species of *Brucella*, *B. melitensis*. Using this security-relevant database, we found that the spectra of this organism from the original run was identified as *B. melitensis* (score 2.39). MALDI-TOF mass spectrometry is not routinely performed on suspected Risk Group 3 agents; however, if this process is inadvertently performed in the future, the security-relevant database would help identify the pathogen sooner. 

Third, the highest score of *Ochrobactrum* spp. by MALDI-TOF mass spectrometry prompts the technologist to consider *Brucella* spp. Similar to our finding with *B. canis*, it has been reported that *B. melitensis* can be misidentified as *O. anthropi* by MALDI-TOF mass spectrometry by using a library lacking *Brucella* (*8*).

## Conclusions

Our investigation shows that humans interacting with dogs from areas to which *B. canis* is endemic are at risk for acquiring human brucellosis (*9*). *B. canis* seropositivity has also been found in dogs within kennels in Canada (*10*). A workup for fever of unknown origin should include a detailed exposure history, including contact with dogs, particularly imported dogs. Laboratory manipulation of *B. canis* isolates from human clinical samples can result in transmission of the organism to laboratory staff. Proactive measures should be taken to minimize risk for exposure to this potential laboratory hazard.
